# Comparative Studies of Genome-Wide Maps of Nucleosomes between Deletion Mutants of *elp3* and *hos2* Genes of *Saccharomyces cerevisiae*

**DOI:** 10.1371/journal.pone.0016372

**Published:** 2011-01-28

**Authors:** Takashi Matsumoto, Choong-Soo Yun, Hirofumi Yoshikawa, Hiromi Nishida

**Affiliations:** 1 Genome Research Center, NODAI Research Institute, Tokyo University of Agriculture, Tokyo, Japan; 2 Agricultural Bioinformatics Research Unit, Graduate School of Agricultural and Life Sciences, University of Tokyo, Tokyo, Japan; 3 Department of Bioscience, Tokyo University of Agriculture, Tokyo, Japan; Duke University, United States of America

## Abstract

In order to elucidate the influence of histone acetylation upon nucleosomal DNA length and nucleosome position, we compared nucleosome maps of the following three yeast strains; strain BY4741 (control), the *elp3* (one of histone acetyltransferase genes) deletion mutant, and the *hos2* (one of histone deactylase genes) deletion mutant of *Saccharomyces cerevisiae*. We sequenced mononucleosomal DNA fragments after treatment with micrococcal nuclease. After mapping the DNA fragments to the genome, we identified the nucleosome positions. We showed that the distributions of the nucleosomal DNA lengths of the control and the *hos2* disruptant were similar. On the other hand, the distribution of the nucleosomal DNA lengths of the *elp3* disruptant shifted toward shorter than that of the control. It strongly suggests that inhibition of Elp3-induced histone acetylation causes the nucleosomal DNA length reduction. Next, we compared the profiles of nucleosome mapping numbers in gene promoter regions between the control and the disruptant. We detected 24 genes with low conservation level of nucleosome positions in promoters between the control and the *elp3* disruptant as well as between the control and the *hos2* disruptant. It indicates that both Elp3-induced acetylation and Hos2-induced deacetylation influence the nucleosome positions in the promoters of those 24 genes. Interestingly, in 19 of the 24 genes, the profiles of nucleosome mapping numbers were similar between the two disruptants.

## Introduction

Eukaryotic genomic DNA is packaged with histone proteins to form chromatin [1], the most fundamental repeating unit of which is the nucleosome [2]. The precise organization of this chromatin is of utmost importance for the maintenance of eukaryotic genomic DNA. Nucleosomes consist of an octamer of histones, around which the DNA is wrapped [3]. Neighboring nucleosomes are separated by unwrapped linker DNA.

Generally, nucleosomal histone proteins are post-translationally modified [4]. Reversible histone acetylation, which is regulated by histone acetyltransferase [5] and deacetylase [6], [7], is one such modification. The acetylation and deacetylation of the core histone tails play an important role in the regulation of transcription [8], [9]. Generally a histone-modifying protein complex consists of a catalytic subunit and the associated subunits. The budding yeast *Saccharomyces cerevisiae* has 62 subunits including 15 histone acetyltransferase catalytic subunits and 12 histone deacetylase catalytic subunits [10].

Although the histone proteins are so conserved among the eukaryotes, the nucleosomal DNA lengths are different among phylogenetically closed ascomycetous yeasts [11]. In addition the fission yeast *Schizosaccharomyces pombe* has the distinct nucleosome positioning mechanism from *Saccharomyces cerevisiae* [12]. Our previous analyses indicated that the distribution of the nucleosomal DNA lengths of the filamentous ascomycete *Aspergillus fumigatus* showed 2 peaks at 135 nt and at 150 nt [13]. On the other hand, the distribution of the nucleosomal DNA lengths of *A. fumigatus* with the hyperacetylated histones induced by the histone deacetylase inhibitor trichostatin A shifted toward longer with a single peak at 168 nt [14], suggesting that hyperacetylation of histones induced to elongate the nucleosomal DNA length.

In order to elucidate the influence of histone acetylation upon the nucleosomal DNA length and the nucleosome position, we compared the nucleosome maps of the following three yeast strains; strain BY4741 (control), the *elp3* (one of histone acetyltransferase genes) disruptant, and the *hos2* (one of histone deactylase genes) disruptant of *Saccharomyces cerevisiae*.

The Elp3 has the highest evolutionary conservation level among the fungal histone modification proteins [10]. The Elp3 is an integral subunit of elongating RNA polymerase II holoenzyme, which is involved in transcription-associated chromatin modification and remodeling [15], [16]. The main acetylation sites of Elp3 are lysine 14 of histone H3 and lysine 8 of histone H4 [17]. The Hos2 has the third highest evolutionary conservation level among the fungal histone modification proteins [10]. The histone deacetylase Hos2 has at least partially overlapping substrate specificities with other histone deacetylases Rpd3 and Hos1 [18]. On the other hand, Hos2 has a different function from Rpd3; Hos2 functions as a gene activator [19], [20].

## Results

### Distribution of nucleosomal DNA lengths

**Figure 1:**
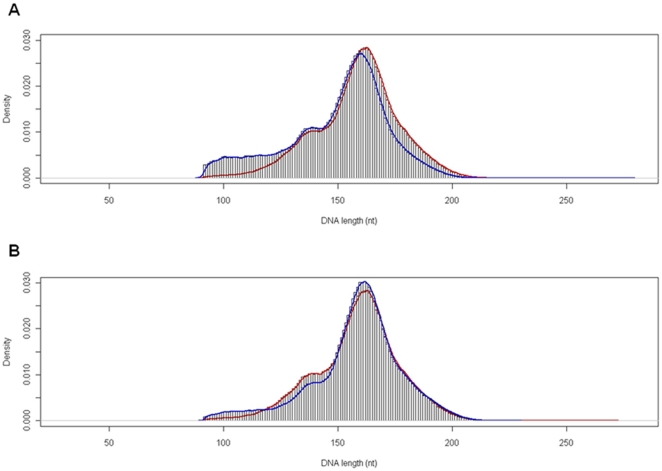
Histograms of the nucleosomal DNA lengths of *Saccharomyces cerevisiae*. (A) Red, the distribution of the nucleosomal DNA lengths of the control (strain BY4741); Blue, that of the *elp3* deletion mutant. (B) Red, the distribution of the nucleosomal DNA lengths of the control (strain BY4741); Blue, that of the *hos2* deletion mutant.

We identified 1578348, 789257, and 2664981 mononucleosomal DNA fragments of strain BY4741, the *elp3* deletion mutant, and the *hos2* deletion mutant, respectively. Those data (the positions of both ends of each DNA fragment) can be downloaded from http://www.iu.a.u-tokyo.ac.jp/~hnishida/data_yeasts.zip. After excluding the completely overlapping DNA fragments, we identified 1522676, 771069, and 2427330 nucleosome positions of strain BY4741, the *elp3* disruptant, and the *hos2* disruptant, respectively. The *elp3* deletion and *hos2* deletion were confirmed using the nucleosome mapping numbers (Fig. S1). The distribution of nucleosomal DNA lengths of strain BY4741 had two peaks at 139 nt (minor) and 163 nt (major) (Fig. 1). The distribution of nucleosomal DNA lengths of the *elp3* disruptant had three peaks at 136 nt (minor), 139 nt (minor), and 160 nt (major) (Fig. 1A). The distribution of nucleosomal DNA lengths of the *hos2* disruptant had two peaks at 141 nt (minor) and 162 nt (major) (Fig. 1B).

### Comparison of conservation levels of nucleosome positions in promoters

**Figure 2:**
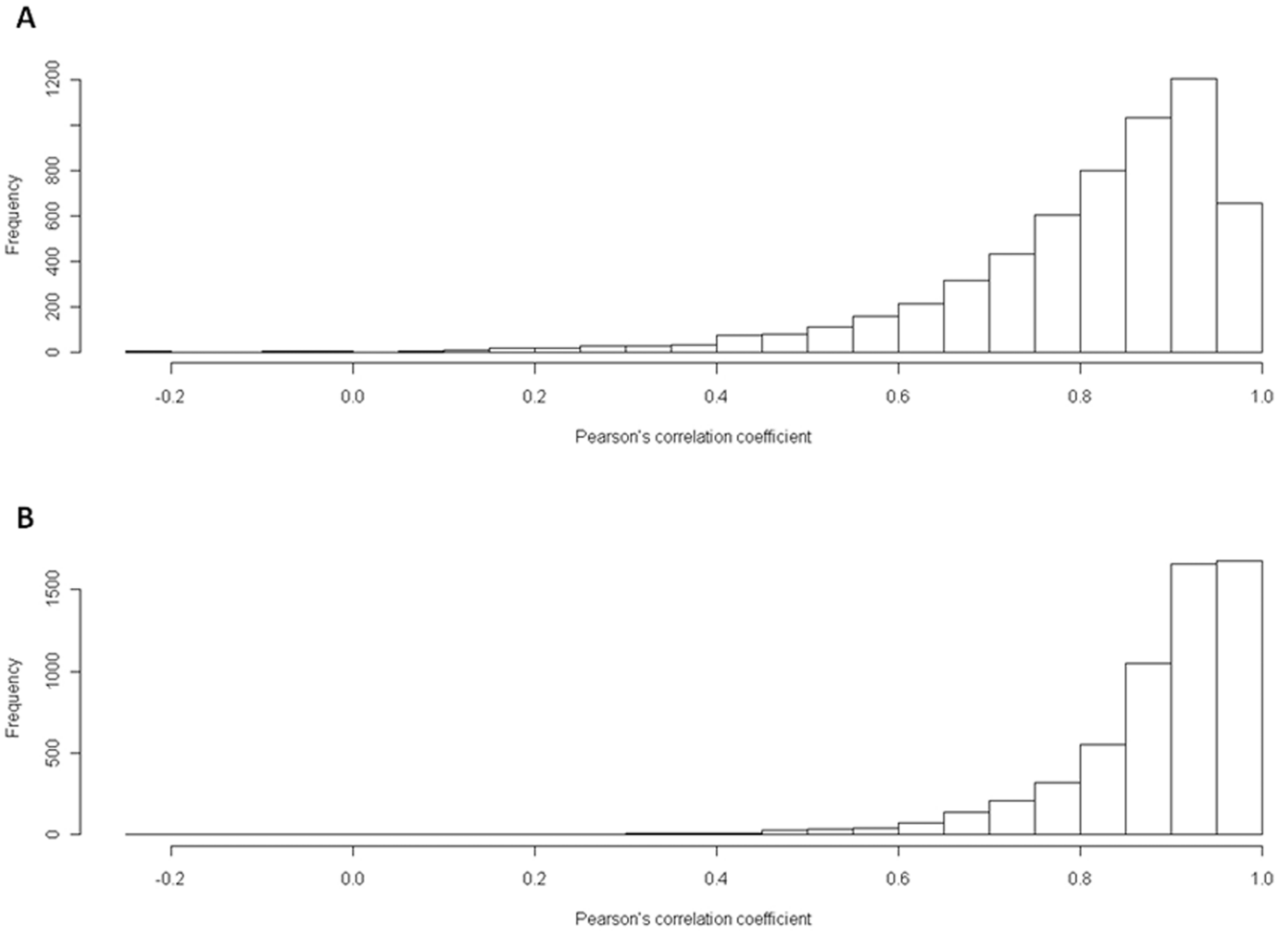
Histograms of the Pearson's correlation coefficients between the profiles of the control and the disruptant nucleosome mapping numbers in the promoters of 5869 protein-coding genes. (A) Pearson's correlation coefficients between the profiles of the control and the *elp3* deletion mutant. (B) Pearson's correlation coefficients between the profiles of the control and the *hos2* deletion mutant.

We calculated the Pearson's correlation coefficients between the profiles of strain BY4741 (control) and the disruptant nucleosome mapping numbers in the promoters of 5869 protein-coding genes. The results were shown in Table S1. The distribution of the Pearson's correlation coefficients between the profiles of the control and the *elp3* disruptant nucleosome mapping numbers was shown in Fig. 2A. That of the Pearson's correlation coefficients between the profiles of the control and the *hos2* disruptant nucleosome mapping numbers was shown in Fig. 2B.

In this study, we used the genes with Pearson's correlation coefficient <0.5 as the genes with low conservation level of nucleosome positions in promoters. Between the control and the *elp3* disruptant, 283 genes had the Pearson's correlation coefficient <0.5. Between the control and the *hos2* disruptant, 53 genes had the Pearson's correlation coefficient <0.5. We detected 24 genes with low conservation level of nucleosome positions in promoters between the control and the *elp3* disruptnat as well as between the control and the *hos2* disruptant (Table S2).

## Discussion

The distribution of nucleosomal DNA lengths of the control *Saccharomyces cerevisiae* (strain BY4741) had two peaks at 139 nt and 163 nt unequally (Fig. 1). The peak at 139 nt is minor and the peak at 163 nt is major. On the other hand, the distribution of nucleosomal DNA lengths of *Aspergillus fumigatus* had two peaks at 135 nt and 150 nt equivalently [13]. The distribution shape of *S. cerevisiae* nucleosomal DNA lengths is so different from that of *A. fumigatus*, suggesting that the difference of those distributions would be applied in the fungal systematics or classification.

The distribution of the nucleosomal DNA lengths of the control is similar to that of the *hos2* disruptant but is different from that of the *elp3* disrutant (Fig. 1AB). The distribution of the nucleosomal DNA lengths of the *elp3* disruptant shifted toward shorter than that of the control, strongly suggesting that inhibition of Elp3-induced histone acetylation causes the nucleosomal DNA length reduction.

In *A. fumigatus*, the hyperacetylation of core histones induced by the histone deacetylase inhibitor trichostatin A causes the nucleosomal DNA length elongation [14]. On the other hand, it seemed that inhibition of histone deacetylation by Hos2 does not influence the nucleosomal DNA length. Thus, the nucleosome map of the *hos2* disruptant of *S. cerevisiae* is consistent with the fact that genome-wide histone acetylation level is not so different between the control and the single gene *hos2* disruptant [18].

Generally the eukaryotes have more conserved nucleosome positions in gene promoters than other regions and the histone modification in promoters plays an important role in the gene regulation [14], [21]–[26]. In order to elucidate the relation between histone acetylation and nucleosome position, we compared the conservation level of nucleosome positions in promoters between the control and the disruptant. In the control and the two disruptants, the distributions of nucleosomal DNA lengths in the promoters were much similar to those of the whole genomes (Fig. S2).

The distributions of the Pearson's correlation coefficients between the profiles of nucleosome mapping numbers in the promoters of the control and the disruptants show that the *elp3* deletion influences the nucleosome positions in the promoters more strongly than the *hos2* deletion (Fig. 2AB). It suggests that the *hos2*-specific histone modification targets are limited. It may be related to the fact that Hos2 has at least partially overlapping substrate specificities with other histone deacetylases Rpd3 and Hos1 [18].

We detected 24 genes with low conservation level of nucleosome positions in promoters between the control and the *elp3* disruptnat as well as between the control and the *hos2* disruptant (Table S2), indicating that both Elp3-induced acetylation and Hos2-induced deacetylation influence the nucleosome positions in the promoters of the 24 genes. Although those 24 genes had the Pearson's correlation coefficient <0.5 between the control and the disruptant, 19 of the 24 genes had the Pearson's correlation coefficient >0.5 between the two disruptants (Table S2). In addition, in order to detect the change in RNA expression of the 24 genes, we performed quantitative RT-PCR. We detected the change in expression of 22 of the 24 genes (Fig. S3). Among the 22 genes, 16 genes were repressed in both the *elp3* and *hos2* deletion mutants (Fig. S3), suggesting that the change in nucleosome positioning induced by *elp3* or *hos2* deletion influenced RNA expression of the 16 genes.

**Figure 3:**
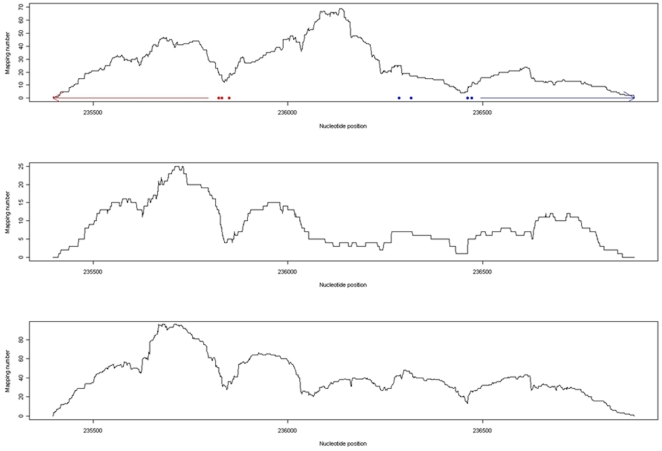
Comparison of mapping numbers of the nucleosomes around *hta2* and *htb2* genes. Top, mapping numbers of the nucleosomes of the control; Middle, mapping numbers of the nucleosomes of the *elp3* deletion mutant; Bottom, mapping numbers of the nucleosomes of the *hos2* deletion mutant. Red arrow indicates the region from the translational start site to the end of *hta2* gene (histone H2A coding). Red dots indicate the transcription start sites of *hta2*. Blue arrow indicates the region from the translational start site to the end of *htb2* gene (histone H2B coding). Blue dots indicate the transcription start sites of *htb2*. Those transcription start sites are based on the data of Miura et al. [31].

Interestingly bidirectional promoter of histone H2A and H2B coding genes (*hta2* and *htb2*) was influenced by both the *elp3* and *hos2* deletions (Table S2, Fig. 3). The profiles of the nucleosome mapping numbers of the *elp3* and *hos2* disruptants were so similar with each other but were different from that of the control (Fig. 3). This is an example of the fact that Elp3 and Hos2 have the same effect on nucleosome positions in the promoters. More works are needed in order to elucidate the mechanism. It is hypothesized that another protein that could be acetylated by Elp3 influences nucleosome positioning.

Among the 53 genes with low conservation level of nucleosome positions in promoters between the control and the *hos2* disruptnat, 24 genes (45%) had also low conservation level between the control and the *elp3* disruptant (Tables S1 and S2). It strongly suggests that Hos2 cooperates with Elp3 for the 24 gene regulations [19], [20]. Our findings suggest that Hos2 functions in not only protein-coding regions [19], [20] but also promoter regions.

## Materials and Methods

### Preparation of mononucleosomal DNA fragments

*Saccharomyces cerevisiae* strains used in this study are EUROSCARF Y00000 (strain BY4741), Y02742 (*elp3* deletion mutant), and Y04561 (*hos2* deletion mutant). These strains were grown in YPD media at 30°C overnight. Each culture was diluted to an absorbance at 600 nm (A_600_) of 0.1/ml into 50 ml of YPD media. These 50 ml cultures were grown at 30°C to an A_600_ of 0.8/ml. Cells were collected, and resuspended in 10 ml of Zymolyase buffer (1 M sorbitol, 50 mM Tris-HCl at pH 7.4 and 10 mM β-mercaptoethanol). Zymolyase-20T (SEIKAGAKU BIOBUSINESS CORPORATION, Tokyo, Japan) was added to a final concentration of 0.25 mg/ml and cells were spheroplasted at 30°C while gently rolling for 30 min. After zymolyase treatment, spheroplasts were collected and resuspended in 2.5 ml of NP buffer (1 M sorbitol, 50 mM NaCl, 10 mM Tris-HCl at pH 7.4, 5 mM MgCl_2_, 1 mM CaCl_2_ and 0.075% Nonidet P40, with freshly added 1 mM β-mercaptoethanol and 500 µM spermidine). Spheroplasts were divided into 7 aliquots of 350 µl, and then micrococcal nuclease (MNase) (Takara Bio Inc., Shiga, Japan) was added at concentrations of 0, 0.05, 0.1, 0.25, 0.5, 0.75 and 1 U per sample. The digestion reactions were incubated at 37°C for 30 min, and were stopped by adding SDS to a final concentration of 1% and EDTA to a final concentration of 10 mM. 5 µl of proteinase K solution (20 mg/ml; QIAGEN Inc., Valencia, CA, USA) was added to each tube, and incubated at 56°C for 1 h. Samples were phenol/chloroform extracted, ethanol precipitated, and treated with RNase (Roche Diagnostics GmbH, Mannheim, Germany). To isolate the mononucleosomal DNA fragments, electrophoresis was carried out on a 2% agarose gel (Fig. S4). We used the mononucleosomal DNA fragments added at concentration of 0.75 U of MNase (Fig. S4, lane 7). The mononucleosomal DNA band was excised and purified using the QIAquick Gel Extraction Kit (QIAGEN Inc.).

### Sequencing and read mapping

The mononucleosomal DNA fragments were prepared for sequencing on a Genome Analyzer II (Illumina, Inc., San Diego, CA, USA). Libraries were generated using Multiplexing Sample Preparation Oligonuclotide Kit (Illumina, Inc.), and sequenced as paired-end 91 bp reads according to the manufacturer's protocols. Using Burrows-Wheeler Aligner [27], sequencing reads were mapped to the genome of *S. cerevisiae* S288C (GenBank accession numbers NC_001133 to NC_001148 and NC_001224). The paired reads that were mapped uniquely in the proper direction, were used for the identification of nucleosome positions.

### Comparison of conservation levels of nucleosome positions in promoters

On the basis of each nucleosomal DNA fragment sequence, nucleosomal mapping numbers were estimated for each nucleotide position [28]. The gene promoter was defined as the region from 1 kb upstream of the translational start site. Pearson's correlation coefficient between the profiles of the control and the disruptant nucleosome mapping numbers at each gene promoter was calculated. Thus, when the profiles are identical, the value is 1.

## Supporting Information

Figure S1 **Mapping numbers of the nucleosomes around** ***elp3*** **and** ***hos2*** **genes.** Right side, region around *elp3*; Left side, region around *hos2*. Top, Strain BY4741 (control); Middle, The *elp3* disruptant; Bottom, The *hos2* disruptant. Arrow indicates the region from the translational start site to the end.
(PPTX)

Figure S2 **Comparison between the distribution of nucleosomal DNA lengths of the whole genome and that in the gene promoters.** (A) Strain BY4741 (control). (B) The *elp3* deletion mutant. (C) The *hos2* deletion mutant. Red, the distribution of the nucleosomal DNA lengths of the whole genome; Blue, that in the gene promoters.
(PPTX)

Figure S3 **Fold change of RNA expression of each gene listed in Table S2.** The following primers were used: TCCGGTGGTAAAGGTGGTAA and GAACCAATTCTCTGGGCGTA (both sequences, from 5′ to 3′) for transcripts of YBL003C; GCTTCTAAATTGGCCGCTTA and GAACCAATTCTCTGGGCGTA for transcripts of YBL002W; TTCTTGGCAAGCATTGACTG and CCCATGGCTGTACCTTTGTT for transcripts of YBR018C; ACCAAGATGCACCGTACCAT and ACCAACTTGGACACGGAAAG for transcripts of YBR048W; ATGCGATCGATTTTTCTGCT and TTAAGGCATTTCCCATCTGC for transcripts of YCR099C; TGGACCCCAAAGAATACGAG and ACAACCGTTCCTGTTGTTCC for transcripts of YDR389W; TGAAAACTTCACAGGGAGAAA and GAAACCATGATTGGGAGACG for transcripts of YDR504C; CCTGGTCTGATCCATGCTTT and ATCATCCGAGGAGGAGAGGT for transcripts of YDR525W-A; CCTGAAAGAACGACCCCATA and CAAAGCGTGCAGAAATCAAA for transcripts of YER185W; GCTGGCCACAGAGAAGAATC and ACGTCGGAGAAGAGCCACTA for transcripts of YFL033C; GGGAAATTCCTGGATCGAAT and AACGTTTTGTTCGTCGGTTC for transcripts of YGR211W; ATCGTCGGAGCTGAAAAAGA and GTTCAATCTGTGGGGCATCT for transcripts of YHR011W; CCAGATGTGCCAACTGTGTC and GCAGCCTCAGTTTGTTCCTT for transcripts of YIL052C; AGCAGGCTCGTCAAGGTAAA and TTACCGATACCTGGCTCACC for transcripts of YLL026W; GCAACATCGTGCTGAGTGAT and CACATCGTCTTTCGGACTCA for transcripts of YLR438C-A; TATGCCCACGTAAACCCATT and CGAAATTGAGTGCACATGCT for transcripts of YLR464W; AGATTGAAAGGTTGCGGATG and CTCTCTTGGCCCCAATCATA for transcripts of YMR032W; ATTCTGCAGCAACCGCTACT and GTTAACGCCGAGTCTTCTGC for transcripts of YMR104C; CAATGCCATGGTCTGTCAAG and TAACCTTGGCAGCTTCGTCT for transcripts of YNL336W; TTATGCCAAGCCCTTAAACG and TTGGGGAAAAGGGTGTCATA for transcripts of YNL269W; ACATCGACCCCAAACTCAAG and AATCCAACCGCAATTGAAAG for transcripts of YNR062C; CCAGTATGTCCCGCAGAAAT and GTTCGCTCGCATAAGTCACA for transcripts of YOR140W; AGGTTTTGTCCGTGGATGAC and CGCCGAATATGTAGCCATTT for transcripts of YOR262W; AGATGAAAAATCGCCTGTGG and CACCTTCGGGTACTTTCCAA for transcripts of YOR356W; and CGGTAGATACGCTGGTGAAGTTTC and TGGAAGATGGAGCAGTGATAACAAC for transcripts of TDH3. Quantification of TDH3 mRNAs were used as control for data normalization [29]. PCR amplification was performed on an ABI PRISM 7300 Real Time PCR System (Applied Biosystems). Expression was assessed by evaluating threshold cycle (Ct) values. We used median of three replicates as representative value. The relative amount of expressed RNA was calculated using Livak and Schmittgen's method [30]. The two genes *YDR504C* and *YER185W* expressions were not determined.
(PPTX)

Figure S4 **Agarose gel electrophoresis of DNA fragments digested by different concentrations of MNase.** Lane 1, DNA size marker; Lane 2, MNase free; Lane 3, 0.05 U of MNase; Lane 4, 0.1 U of MNase; Lane 5, 0.25 U of MNase; Lane 6, 0.5 U of MNase; Lane 7, 0.75 U of MNase; Lane 8, 1 U of MNase. Arrows indicate the location of mononucleosomal DNA fragments.
(PPTX)

Table S1 **Pearson's correlation coefficient between the profiles of the control and the disruptant nucleosome mapping numbers in each gene promoter.**
(XLS)

Table S2 **Genes with low conservation level of nucleosome positions in promoters.**
(DOCX)
