# Supplementary figures and images for: Comparative Studies of Genome-Wide Maps of Nucleosomes between Deletion Mutants of elp3 and hos2 Genes of Saccharomyces cerevisiae
Source: PLoS One. 2011 Jan 28;6(1):e16372. doi: 10.1371/journal.pone.0016372 (PMC3030569; doi:10.1371/journal.pone.0016372)

## Slide 1
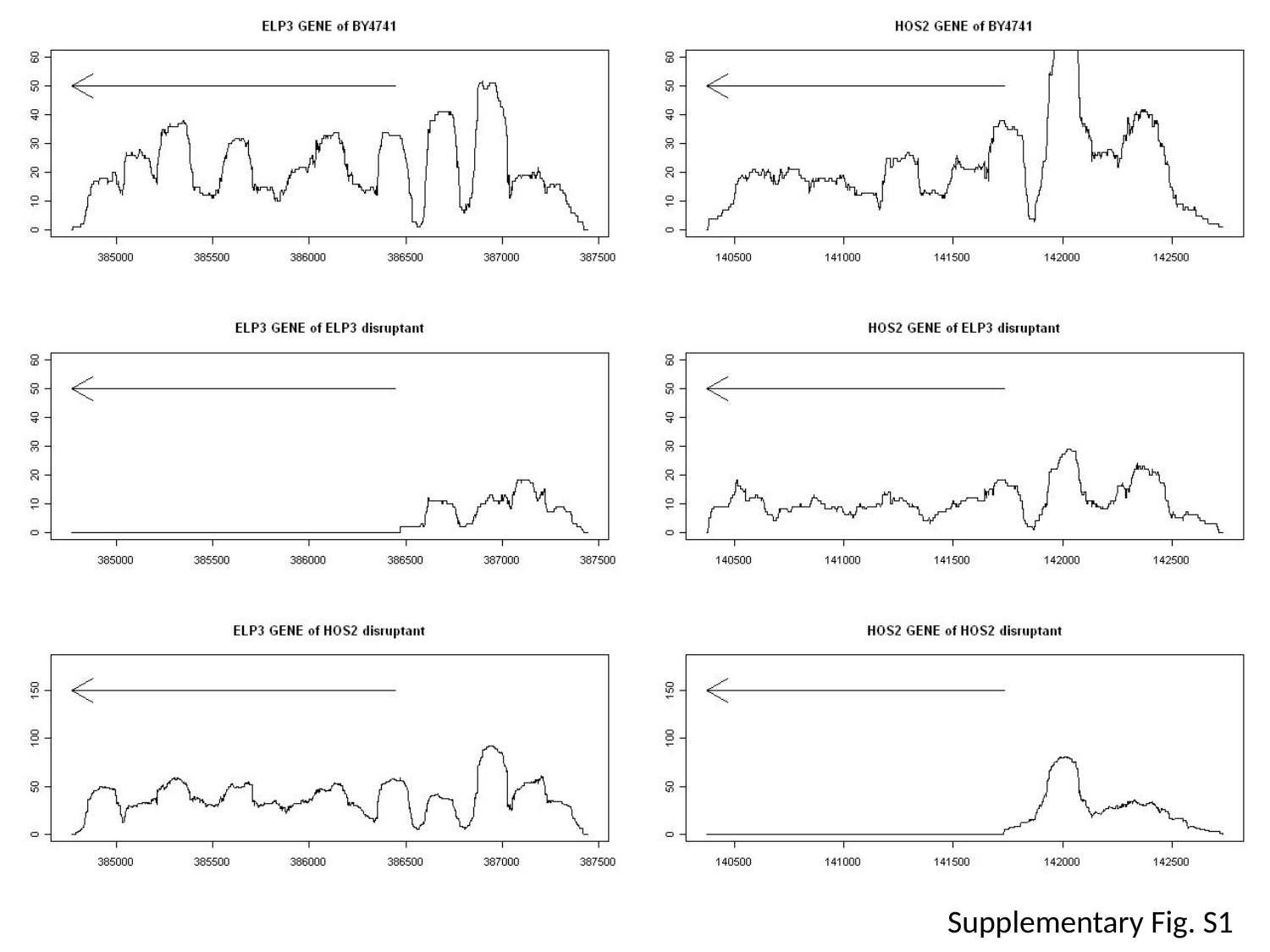

Supplementary Fig. S1

Supplement: Figure S1 — Mapping numbers of the nucleosomes around elp3 and hos2 genes. Right side, region around elp3; Left side, region around hos2. Top, Strain BY4741 (control); Middle, The elp3 disruptant; Bottom, The hos2 disruptant. Arrow indicates the region from the translational start site to the end. (PPTX) [file pone.0016372.s001.pptx]

## Slide 1
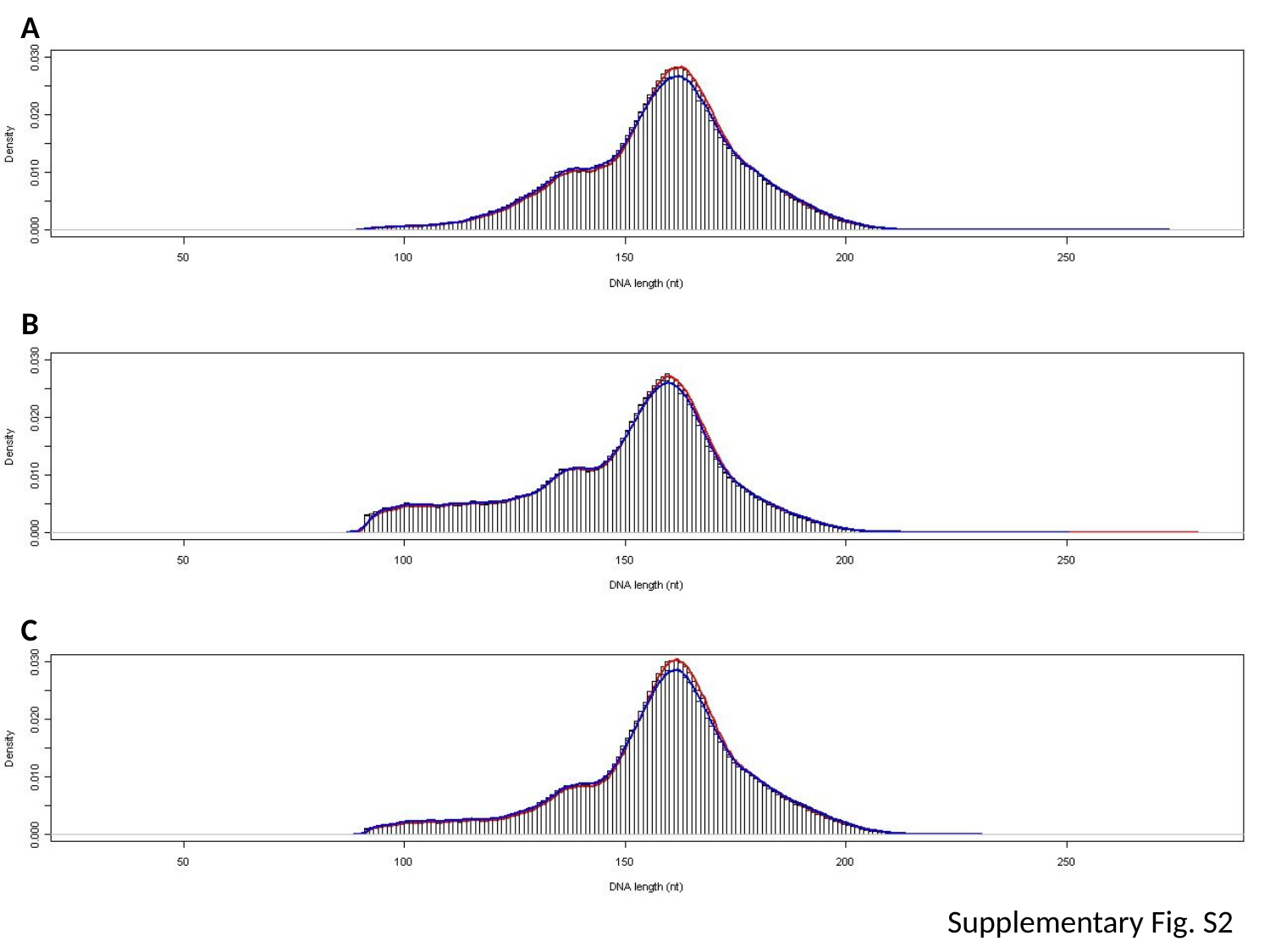

A
B
C
Supplementary Fig. S2

Supplement: Figure S2 — Comparison between the distribution of nucleosomal DNA lengths of the whole genome and that in the gene promoters. (A) Strain BY4741 (control). (B) The elp3 deletion mutant. (C) The hos2 deletion mutant. Red, the distribution of the nucleosomal DNA lengths of the whole genome; Blue, that in the gene promoters. (PPTX) [file pone.0016372.s002.pptx]

## Slide 1
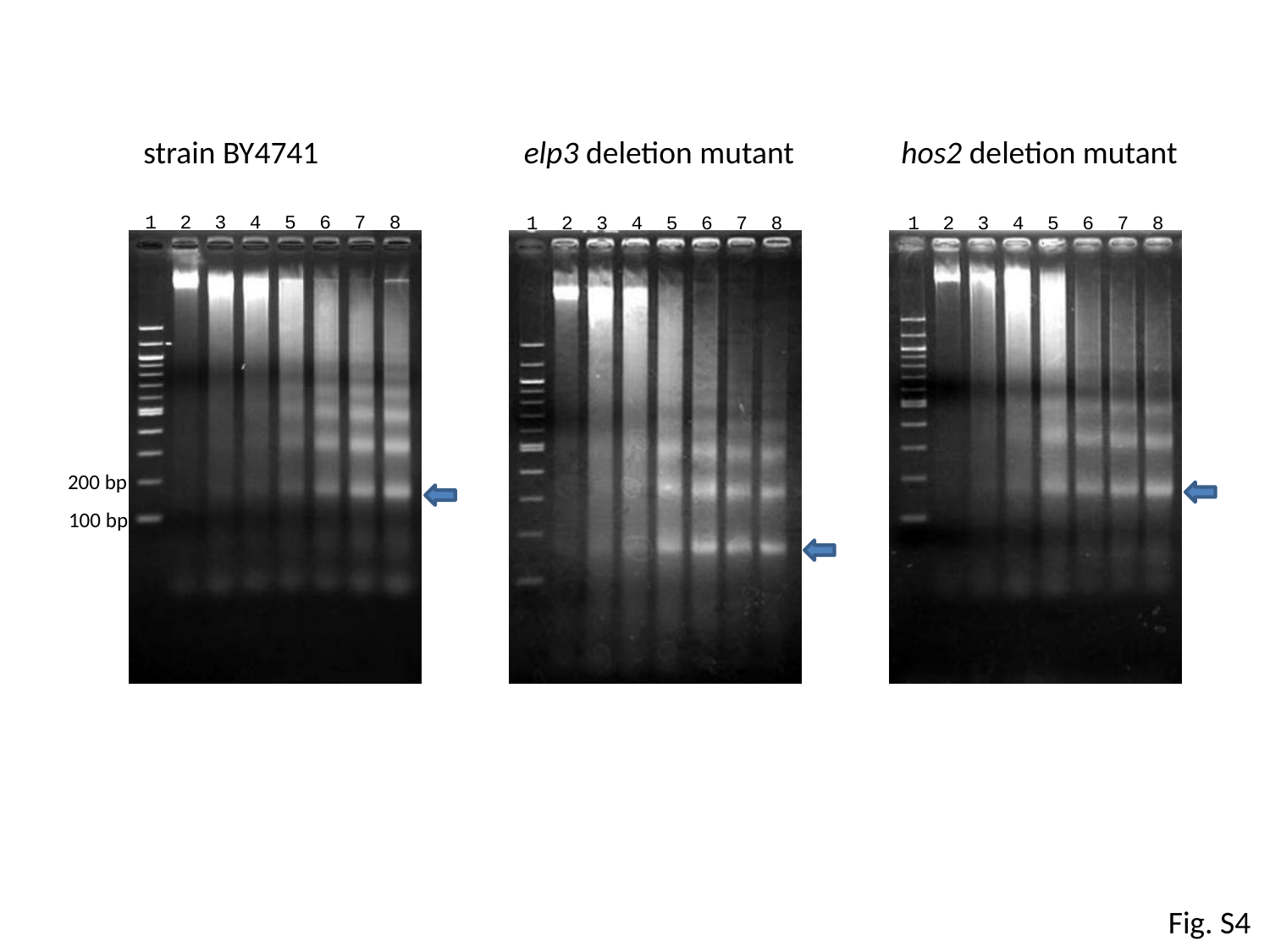

strain BY4741
elp3 deletion mutant
hos2 deletion mutant
1 2 3 4 5 6 7 8
1 2 3 4 5 6 7 8
1 2 3 4 5 6 7 8
200 bp
100 bp
Fig. S4

Supplement: Figure S4 — Agarose gel electrophoresis of DNA fragments digested by different concentrations of MNase. Lane 1, DNA size marker; Lane 2, MNase free; Lane 3, 0.05 U of MNase; Lane 4, 0.1 U of MNase; Lane 5, 0.25 U of MNase; Lane 6, 0.5 U of MNase; Lane 7, 0.75 U of MNase; Lane 8, 1 U of MNase. Arrows indicate the location of mononucleosomal DNA fragments. (PPTX) [file pone.0016372.s004.pptx]
