# Supplementary material for: Comparative Studies of Genome-Wide Maps of Nucleosomes between Deletion Mutants of elp3 and hos2 Genes of Saccharomyces cerevisiae
Source: PLoS One. 2011 Jan 28;6(1):e16372. doi: 10.1371/journal.pone.0016372 (PMC3030569; doi:10.1371/journal.pone.0016372)
Supplement: Figure S3 — Fold change of RNA expression of each gene listed in Table S2. The following primers were used: TCCGGTGGTAAAGGTGGTAA and GAACCAATTCTCTGGGCGTA (both sequences, from 5′ to 3′) for transcripts of YBL003C; GCTTCTAAATTGGCCGCTTA and GAACCAATTCTCTGGGCGTA for transcripts of YBL002W; TTCTTGGCAAGCATTGACTG and CCCATGGCTGTACCTTTGTT for transcripts of YBR018C; ACCAAGATGCACCGTACCAT and ACCAACTTGGACACGGAAAG for transcripts of YBR048W; ATGCGATCGATTTTTCTGCT and TTAAGGCATTTCCCATCTGC for transcripts of YCR099C; TGGACCCCAAAGAATACGAG and ACAACCGTTCCTGTTGTTCC for transcripts of YDR389W; TGAAAACTTCACAGGGAGAAA and GAAACCATGATTGGGAGACG for transcripts of YDR504C; CCTGGTCTGATCCATGCTTT and ATCATCCGAGGAGGAGAGGT for transcripts of YDR525W-A; CCTGAAAGAACGACCCCATA and CAAAGCGTGCAGAAATCAAA for transcripts of YER185W; GCTGGCCACAGAGAAGAATC and ACGTCGGAGAAGAGCCACTA for transcripts of YFL033C; GGGAAATTCCTGGATCGAAT and AACGTTTTGTTCGTCGGTTC for transcripts of YGR211W; ATCGTCGGAGCTGAAAAAGA and GTTCAATCTGTGGGGCATCT for transcripts of YHR011W; CCAGATGTGCCAACTGTGTC and GCAGCCTCAGTTTGTTCCTT for transcripts of YIL052C; AGCAGGCTCGTCAAGGTAAA and TTACCGATACCTGGCTCACC for transcripts of YLL026W; GCAACATCGTGCTGAGTGAT and CACATCGTCTTTCGGACTCA for transcripts of YLR438C-A; TATGCCCACGTAAACCCATT and CGAAATTGAGTGCACATGCT for transcripts of YLR464W; AGATTGAAAGGTTGCGGATG and CTCTCTTGGCCCCAATCATA for transcripts of YMR032W; ATTCTGCAGCAACCGCTACT and GTTAACGCCGAGTCTTCTGC for transcripts of YMR104C; CAATGCCATGGTCTGTCAAG and TAACCTTGGCAGCTTCGTCT for transcripts of YNL336W; TTATGCCAAGCCCTTAAACG and TTGGGGAAAAGGGTGTCATA for transcripts of YNL269W; ACATCGACCCCAAACTCAAG and AATCCAACCGCAATTGAAAG for transcripts of YNR062C; CCAGTATGTCCCGCAGAAAT and GTTCGCTCGCATAAGTCACA for transcripts of YOR140W; AGGTTTTGTCCGTGGATGAC and CGCCGAATATGTAGCCATTT for transcripts of YOR262W; AGATGAAAAATCGCCTGTGG and CACCTTCGGGTACTTTCCAA for transcripts of YOR356W; and CGGTAGATACGCTGGTGAAGTTTC and TGGAAGATGGAGCAGTGATAACAAC for transcripts of TDH3. Quantif [file pone.0016372.s003.pptx]

## Slide 1
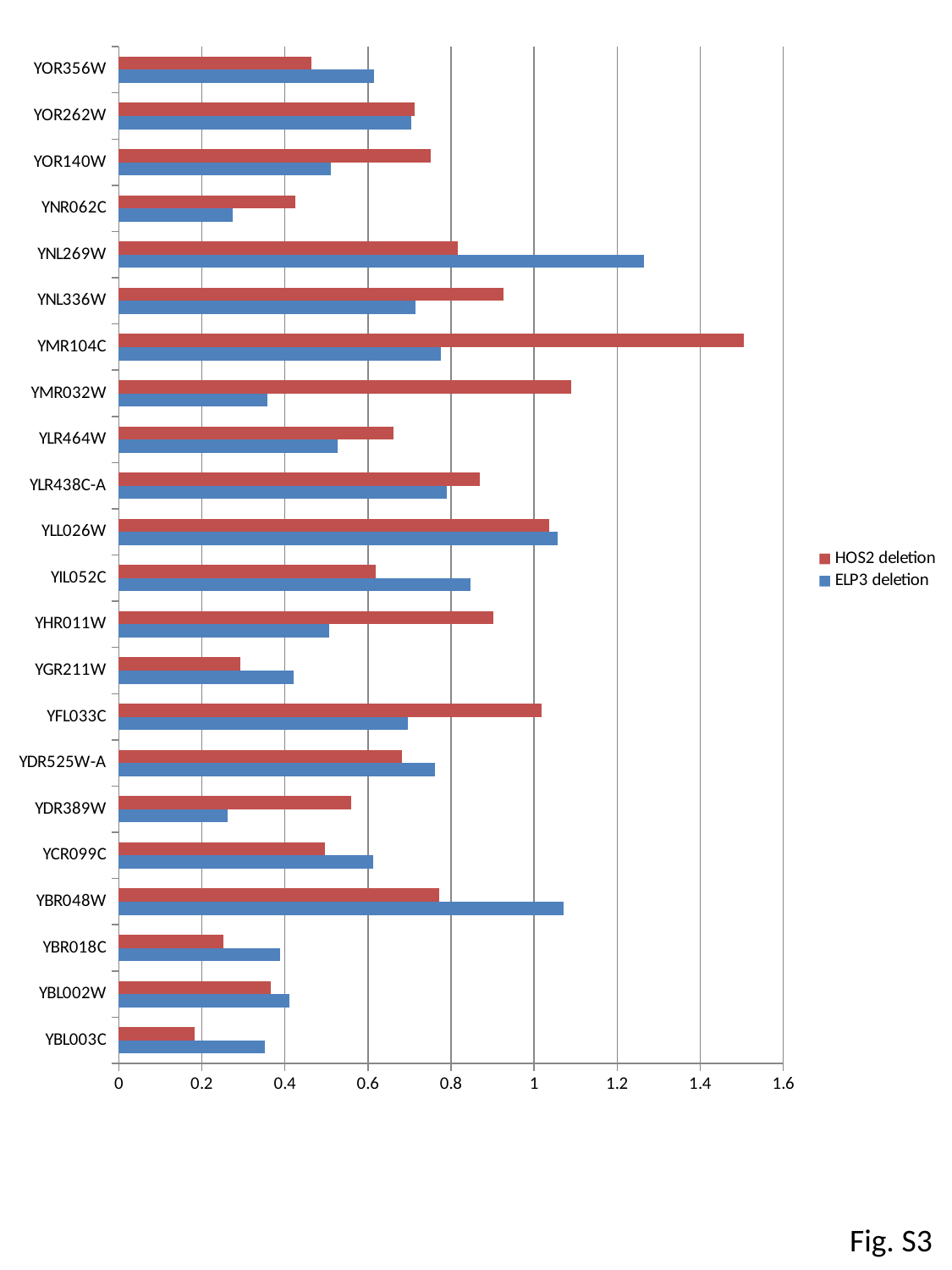

### Chart
| Category | ELP3 deletion | HOS2 deletion |
|---|---|---|
| YBL003C | 0.35230576796417784 | 0.1825166519836446 |
| YBL002W | 0.41148164991629443 | 0.3654130340478914 |
| YBR018C | 0.38920442019128665 | 0.252437824399172 |
| YBR048W | 1.070882357083502 | 0.7705711083584071 |
| YCR099C | 0.6123803942987192 | 0.4957896295680768 |
| YDR389W | 0.262884320248502 | 0.5598064443271198 |
| YDR525W-A | 0.7609639425904693 | 0.6816958001954825 |
| YFL033C | 0.6963574708398871 | 1.018891196616721 |
| YGR211W | 0.4214402466019774 | 0.29264022610630863 |
| YHR011W | 0.5064880023179306 | 0.9028135645871879 |
| YIL052C | 0.8460413022415976 | 0.6191667911087004 |
| YLL026W | 1.0566517694976796 | 1.0364855474984684 |
| YLR438C-A | 0.7899865522704378 | 0.8690433204285092 |
| YLR464W | 0.5267170259542823 | 0.6609440245698783 |
| YMR032W | 0.35843911834667774 | 1.0899031944061226 |
| YMR104C | 0.7751245221017218 | 1.5044122927611907 |
| YNL336W | 0.7152899120415339 | 0.9254327113606585 |
| YNL269W | 1.263652690958282 | 0.815241839552771 |
| YNR062C | 0.2741999094873276 | 0.42484249956932424 |
| YOR140W | 0.5115332768534896 | 0.7511640897070004 |
| YOR262W | 0.7039769025532385 | 0.7118770517350156 |
| YOR356W | 0.6151883125021915 | 0.4629088340247031 |Fig. S3
