# Supplementary material for: Comparative Studies of Genome-Wide Maps of Nucleosomes between Deletion Mutants of elp3 and hos2 Genes of Saccharomyces cerevisiae
Source: PLoS One. 2011 Jan 28;6(1):e16372. doi: 10.1371/journal.pone.0016372 (PMC3030569; doi:10.1371/journal.pone.0016372)
Supplement: Table S2 — Genes with low conservation level of nucleosome positions in promoters. (DOCX) [file pone.0016372.s006.docx]

| Table S2. Genes with low conservation level of nucleosome positions in promoters. | | | | | | | | |
| --- | --- | --- | --- | --- | --- | --- | --- | --- |
| Chromosome | Correlation coefficient between the control and the *elp3* disruptant | Correlation coefficient between the control and the *hos2* disruptant | Correlation coefficient between the *elp3* and the *hos2* disruptants | Translational start site | Transcription direction | Locus tag | Gene name | Function |
| chr02 | 0.249607174 | 0.473673107 | 0.7721589 | 235795 | - | YBL003C | HTA2 | histone H2A |
| chr02 | 0.348375767 | 0.456574762 | 0.925312 | 236495 | + | YBL002W | HTB2 | histone H2B |
| chr02 | 0.167687998 | 0.498226441 | 0.4466425 | 275527 | - | YBR018C | GAL7 | galactose-1-phosphate uridyl transferase |
| chr02 | 0.236843537 | 0.48510247 | 0.7772281 | 332829 | + | YBR048W | RPS11B | component of the small ribosomal subunit |
| chr03 | -0.016805523 | 0.487126621 | 0.4006219 | 301296 | - | YCR099C |  | unknown |
| chr04 | 0.085009851 | 0.232139365 | 0.7189923 | 1252529 | + | YDR389W | SAC7 | GTPase activating protein for Rho1 |
| chr04 | 0.152408275 | 0.28069126 | 0.5575123 | 1456686 | - | YDR504C | SPG3 | protein required for survival at high temperature during stationary phase |
| chr04 | 0.055918124 | 0.486328976 | 0.5588501 | 1490588 | + | YDR525W-A | SNA2 | unknown |
| chr05 | 0.443413247 | 0.410937945 | 0.8950202 | 559449 | + | YER185W | PUG1 | plasma membrane protein involved in protoporphyrin uptake |
| chr06 | 0.408998973 | 0.400870707 | 0.04896357 | 74425 | - | YFL033C | RIM15 | glucose-repressible protein kinase |
| chr07 | 0.11172062 | 0.206927302 | 0.7238332 | 915246 | + | YGR211W | ZPR1 | essential protein with two zinc fingers |
| chr08 | 0.151686283 | 0.223618366 | 0.8327685 | 127774 | + | YHR011W | DIA4 | probable mitochondrial seryl-tRNA synthetase |
| chr09 | 0.422569353 | 0.446417635 | 0.6548925 | 257061 | - | YIL052C | RPL34B | component of the large ribosomal subunit |
| chr12 | 0.194538514 | 0.490307343 | 0.8172198 | 88622 | + | YLL026W | HSP104 | heat shock protein that cooperates with Ydj1p and Ssa1p |
| chr12 | 0.380423431 | 0.303213268 | 0.847854 | 1014175 | - | YLR438C-A | LSM3 | U6 snRNA-associated Sm-like protein |
| chr12 | 0.07769072 | 0.340055324 | 0.7438567 | 1066570 | + | YLR464W |  | unknown |
| chr13 | 0.38055936 | 0.496357301 | 0.5092175 | 335297 | + | YMR032W | HOF1 | bud neck-localized, SH3 domain-containing protein required for cytokinesis |
| chr13 | 0.101118688 | 0.483732376 | 0.8159712 | 475452 | - | YMR104C | YPK2 | protein kinase |
| chr14 | 0.169243455 | 0.31430664 | 0.1470152 | 8330 | + | YNL336W | COS1 | unknown |
| chr14 | 0.418800862 | 0.472949337 | 0.6864377 | 137700 | + | YNL269W | BSC4 | unknown |
| chr14 | 0.390506697 | 0.450747268 | 0.07042813 | 745343 | - | YNR062C |  | putative membrane protein |
| chr15 | 0.470409741 | 0.453495316 | 0.5922247 | 586982 | + | YOR140W | SFL1 | transcriptional repressor and activator |
| chr15 | 0.41110728 | 0.408744637 | 0.738176 | 817291 | + | YOR262W |  | unknown |
| chr15 | 0.188881962 | 0.356846187 | 0.8161629 | 1007219 | + | YOR356W |  | mitochondrial protein with similarity to flavoprotein-type oxidoreductases |
|  |  |  |  |  |  |  |  |  |
